# Where does selective laser trabeculoplasty stand now? A review

**DOI:** 10.1186/s40662-016-0041-y

**Published:** 2016-04-05

**Authors:** Myrjam De Keyser, Maya De Belder, Simon De Belder, Veva De Groot

**Affiliations:** Department of Medicine, University of Antwerp, Universiteitsplein 1, B-2610 Antwerp, Belgium; Medipolis, Boomsesteenweg 223, B-2610 Antwerp, Belgium; Faculty of Psychology and Education, Department of Experimental Psychology, Ghent University, Henri Dunantlaan 2, B-9000 Ghent, Belgium; Department of Ophthalmology, University Hospital Antwerp, Wilrijkstraat 10, B-2650 Edegem, Belgium

**Keywords:** Glaucoma, Selective laser trabeculoplasty, Review, Open angle glaucoma, Glaucoma treatment, Ocular hypertension

## Abstract

**Background:**

Chronic treatment of glaucoma can present a challenge in patients who lack the means and/or the discipline to use daily glaucoma medication. We wondered if selective laser trabeculoplasty (SLT) could be a useful alternative.

**Methods:**

Inclusion criteria: controlled trials comparing efficacy of SLT in adult patients with any form of open angle glaucoma or ocular hypertension and case reports on side effects of SLT. Two recent meta-analyses identified eight randomized clinical trials (RCTs) comparing the effect of SLT with medication (prostaglandin analogs) and with argon laser trabeculoplasty (ALT). We took these eight RCTs as reference base and calculated their success rates where they were not given. Other articles were added to elaborate on technique and side effects.

**Results:**

Mean intraocular pressure (IOP) reduction after SLT was 3.8–8.0 mmHg after 6 months to 1 year. Mean success rate of SLT at 6 months to 1 year is 55–82 %. Higher IOP before laser predicts a higher IOP-lowering effect. In terms of mean IOP reduction, reduction in number of medications and treatment success, the effect of SLT was found to show no clinically relevant difference from that of contemporary medication (prostaglandin analogs) and from ALT.

**Conclusions:**

The evidence indicates that SLT is an efficacious primary or adjunctive therapy for treating glaucoma.

## Background

Glaucoma is a multifactorial disease [1, 2] characterized by damage to the optic nerve, often caused by an elevated intraocular pressure (IOP). Worldwide, it is the second leading cause of blindness, following cataract. It is predicted that by 2020, 79.6 million people will have glaucoma [3], of which 74 % will be open angle glaucoma (OAG) [4, 5].

The aim of glaucoma therapy is lowering the IOP in order to slow down glaucoma progression. Currently, this goal can be achieved by medication, laser treatment or surgery [6]. Eye drops are typically the first line of therapy for glaucoma. However, topical agents and preservatives in glaucoma medication can produce local and systemic side effects [1, 4, 5, 7]. Patients must also tolerate repeat application of drugs and on-going medical costs [8].

Adherence poses a problem [9]. At best, treatment adherence in chronic diseases is estimated to be 75 % [6, 10]. More complex dosing regimens, as common in glaucoma, result in poorer adherence [4, 8, 9, 11]. Administering topical medications is often problematic for elderly patients who need assistance to administer the drops or miss the eye and waste expensive medication [12]. Not properly using the prescribed drops is a major cause of visual loss in patients with glaucoma [12].

As an alternative to eye drops, argon laser trabeculoplasty (ALT) [13, 14] has proven to be a useful treatment. ALT produces much coagulative damage on biomicroscopy [15] and probably works mechanically through shrinking the trabecular meshwork (TM) tissue at the coagulation site and opening up the adjacent tissue. However, Bylsma et al. showed that ALT also activates a cellular mechanism that stimulates TM cell division, rejuvenating the meshwork [16]. Secretion of interleukins after ALT has also been demonstrated, stimulating remodelling of the extracellular matrix and lowering resistance at the TM [17].

Long-term follow-up showed that after a few years, 68–95 % of patients returned to a higher IOP. Retreatment with ALT was found to be ineffective [12, 18, 19].

Selective laser trabeculoplasty (SLT) was launched in 1998 by Latina et al. [20]. A frequency-doubled, Q-switched, 532 nm Nd:YAG laser is used with pulse duration of 3 nsec and a spot size of 400 μm. This pulse duration is too short for melanin to convert the electromagnetic energy to thermal energy, which means that no heat is generated. The total energy per μm of spotsize in SLT is more than 6500 times lower than in ALT, which provides a theoretical basis to consider SLT as a repeatable treatment for lowering IOP.

In our public hospital, we have a ‘difficult’ population; there is a tendency for irregular follow up examinations (no show) because of lack of means and mostly of discipline to use glaucoma medication, which compromises therapy. Laser could be a non-invasive treatment that can partly solve these problems. The question our patients always ask when we try to motivate them to have SLT done is: ‘What are my chances that this laser will work and what problems can I expect from this laser?’ This article sets out to answer those questions in terms of success rate and side effects of SLT.

## Methods

### Literature search and trial selection

Studies were identified and retrieved through a systematic search of PubMed, Web of Science and Google Scholar. Keywords used in identifying relevant research included ‘selective laser trabeculoplasty’, ‘SLT’, ‘glaucoma treatment’, ‘ocular hypertension’ and ‘side effects of selective laser trabeculoplasty’.

Inclusion criteria: retrospective and prospective comparative controlled trials studying the effect of SLT in adult patients with any form of OAG or ocular hypertension (OHT), with or without medication. We also included case-reports on side effects of SLT.

Exclusion criteria: studies in which SLT was used as adjunct for glaucoma operations.

Recently, two meta-analyses were published on SLT. A meta-analysis by Li et al. [8] identified four randomized clinical trials (RCTs) comparing the effect of SLT with medication [21–24]. The meta-analysis of Wong et al. [25] included these four RCTs and four additional RCTs, that compared SLT to ALT [26–29]. These eight RCTs were taken as reference base for our calculations. Other articles were used to elaborate on technique and side effects.

The information on author, mean age, sample size, type of glaucoma, and study design were extracted. We calculated the success rate if it was not given in the article and when enough data were present to make this calculation [21, 22, 26]. Definition of success was a 20 % lowering of IOP compared to baseline IOP. Where mentioned, the incidence of transient post-laser IOP spikes, redness, anterior chamber reaction, discomfort or other side effects were recorded.

## Review

### Mechanism

Cvenkel et al. applied ALT to a part of the eye, and SLT to another part of the same eye in three patients that were planned to get an enucleation [15]. After the enucleation, the anterior segments of the eyes were dissected and examined. Light microscopy and transmission electron microscopy showed much less structural changes after SLT than after ALT. Damaging the pigmented cells of the TM without mechanical or thermic effect by SLT seemed to be enough to enhance the outflow through the TM. This may be a sign that SLT works more at a cellular level, through stimulation of the formation of healthy TM tissue, like ALT [16, 17, 30]. Detorakis et al. also found arguments for a biochemical mechanism with release of cytokines after laser that possibly attract macrophages and stimulate phagocytosis of debris in the TM [31]. Both cellular and biomechanical mechanisms can enhance the outflow capacity of the TM with minimal damage to the tissue [15, 32].

### Characteristics of intervention

In the eight reference RCTs of Wong et al. [25] and Li et al. [8], 45–102 SLT applications were applied, with a spot size of 400 μm and power between 0.47 and 1.7 mJ, with each spot lasting 3 ns. See also Table 1.

**Table 1:** Baseline characteristics of randomized clinical trials using SLT

Author, year	Design, location	Type of glaucoma	# eyes after SLT	Treatment in control arm	Mean follow-up (months)	Mean age (years)	SLT group baseline IOP (mmHg)	Control group baseline IOP (mmHg)	Characteristics SLT (extent, number spots, power)
Lai, 2004 [22]	SC, China	POAG, OHT	29	medication (not specified)	60	51.9 ± 14.7	26.8 ± 5.6	26.2 ± 4.2	360°, 100, 1.0 ± 0.1 mJ
Nagar, 2005 [23]	MC, UK	POAG, OHT, PEX, PDS	128	medication (lat)	10.3	63	NA	NA	90° (25–30 spots), 180° (48–53 spots), 360° (93–102 spots), 0.8-1.7 mJ
Nagar, 2009 [24]	SC, UK	POAG, OHT	20	medication (lat)	4–6	66.4	26.1 ± 4.0	22.8 ± 4.5	360°, 100 ± 5, 0.8–1.4 mJ
Katz, 2012 [21]	MC, USA	POAG, OHT	67	medication (prost)	6–12	NA	25.0 ± 2.2 (4–6 mth group), 24.5 ± 2.1 (9–12 mth group)	24.5 ± 2.2 (4–6 mth group), 24.7 ± 2.4 (9–12 mth group)	360°, 100, 0.8–1.2 mJ
Bovell, 2011 [26]	SC, Canada	POAG, PEX, PDS, mix mech, others	89	ALT	60	69.7 ± 10.52	23.8 ± 4.9	23.48 ± 4.21	180°, 50, 0.47–1.5 mJ
Liu, 2012 [27]	SC, Canada	POAG, OHT, PEX, PDS, NTG, juv OAG, mix mech	20	ALT	37	48.7 ± 9.4	19.1 ± 4.5	21.9 ± 4.4	180°, 45–55, 0.7–0.8 mJ
Rosenfeld, 2012 [28]	SC, Israel	POAG, OHT, PEX, PDS	22	ALT	12	71.95	25.36 ± 1.83	25.11 ± 2.16	180°, 50–70, 0.8–1.2 mJ
Kent, 2013 [29]	MC, Canada	PEX	37	ALT	6	72.9 ± 9.86	23.1 ± 4.22	25.2 ± 4.87	180°, 53 ± 3.75, 0.6 mJ

*Abbreviations*: *SC* = single centre; *MC* = multicentre; *NA* = not available; *POAG* = primary open angle glaucoma; *OHT* = ocular hypertension; *PDS* = pigment dispersion syndrome; *PEX* = pseudoexfoliation syndrome; *NTG* = normal tension glaucoma; *juv* = juvenile glaucoma; *mix mech* = mixed mechanism glaucoma; *lat* = latanoprost; *prost* = prostaglandin analog

In the 2005 study, Nagar et al. [23] randomized patients to 90, 180, and 360° treatment. The 90° had a very low success rate at a mean of 10.3 months (34 %). Although there was no statistically significant difference in outcome after 180° or 360° of SLT treatment, success rates were better with 360°. In their trial, Bovell et al. [26], Liu et al. [27], Rosenfeld et al. [28], and Kent et al. [29] chose to treat 180° of the TM. Lai et al. [22], Nagar et al. [23], and Katz et al. [21] treated 360°. Katz et al. started by treating 360° and performed a 180° treatment when a second SLT was needed during follow up.

The energy level used is titrated to the degree of trabecular pigmentation. The greater the pigmentation, the less energy is required. To determine the optimal energy level, the power setting is initially set at 0.6–0.9 mJ and the energy level is increased by 0.1 mJ until bubble formation (champagne bubbles) is observed. If bubble formation is already noted at the initial energy level, the laser energy is reduced by 0.1 mJ [20]. An energy level just below that of bubble formation is then maintained. In their population of pseudoexfoliative syndrome (PEX) patients, Kent et al. used a mean energy level of 0.6 mJ [29] for their population of pseudoexfoliation glaucoma patients.

Ayala et al. [33] found that higher energy creates a longer IOP lowering effect after SLT and Lee et al. [34] also suggested using a higher energy density (number of spots multiplied by mean energy) to improve SLT outcome. On the other hand, more energy and more spots may be associated with more side effects [23] and an increased risk of corneal oedema [35], so caution has to be taken.

It may be important to bring in mind that the energy used in SLT is much lower than in ALT. SLT classically uses around 0,9 mJ of energy [23–25], whereas ALT uses around 50 mJ (500 mW for 0.1 s) [26–28]. This makes the energy used by SLT 55 times smaller than in ALT. The laser spot used in SLT has a diameter of 400 μm [23–25] or an area of 0.13 mm^2^. ALT uses a spot of 50 μm [26–28] or an area of 0.002 mm^2^. The energy in ALT is thus aimed at an area 64 times smaller than in SLT. This means that the energy per mm^2^ of tissue is 3520 times smaller in SLT than in ALT.

### Comparison of SLT to medication

The effectiveness of SLT compared to contemporary medication (prostaglandin analogs) was investigated in a large meta-analysis by Li et al. [8]. Five prospective studies were included in their study, four randomized and one non-randomized. Wong et al. identified the same four randomized RCTs. We included these four studies, one published by Lai et al. [22], two by Nagar et al. [23, 24], and one by Katz et al. [21]. Two of them were single centre, while the others were multicentre trials, see Table 1. The number of patients treated with SLT ranged from 20 to 67; the largest trial by Nagar et al. included 128 eyes [23]. Mean follow up time was short: 4 to 12 months. Lai [22] and Bovell [26] had a follow up time of 5 years. The study of Nagar [23] mentioned a range of follow up from 1 to 12 months. In this study, patients were randomized to medication and 90°, 180°, and 360° of SLT. The second trial of Nagar et al. [24] was designed to compare the effect of reduction of IOP fluctuation between SLT and latanoprost, with a mean follow up of 4 to 6 months. This study showed a significantly higher baseline IOP in the latanoprost group, which was adjusted for calculation of the IOP fluctuation. Baseline IOP was above 24.5 mmHg in three of the four RCTs (not available in Nagar [23]). No significant differences in IOP lowering effect could be found between the medication and the SLT groups.

### Comparison of SLT to ALT

Wong et al. [25] conducted a meta-analysis involving all studies on SLT effect in patients with OAG or OHT. They included primary open angle glaucoma (POAG), PEX, pigment dispersion syndrome (PDS), uveitis glaucoma, juvenile glaucoma, steroid-induced glaucoma, and normal tension glaucoma. Four RCTs comparing the efficacy of SLT and ALT were identified. These studies were conducted between 2011 and 2013, which included one multicentre and 3 single centre trials. Baseline characteristics are shown in Table 1. The number of patients treated was again mostly limited (20–37) with one larger study (89 patients) by Bovell et al. [26]. Liu et al. focused on younger patients, with a mean age of 48.7 years and a significantly lower baseline IOP (19.1 mmHg). Rosenfeld et al. [28] recruited patients that underwent successful cataract extraction 3–6 months before the study commenced. Kent et al. [29] limited their study to patients with PEX. All studies concluded that the IOP-lowering effect of SLT was comparable to ALT.

### Success rate

The IOP reduction varied from 3.8 to 8.0 mmHg at 6 months to 1 year after SLT (see Table 2). The lowest reduction of 3.8 mmHg was found in the population of Liu et al. [27]. This group started with a low mean baseline IOP (19.1 mmHg). As a higher baseline IOP is predictive of a greater IOP decrease, a lower response could be expected.

**Table 2:** Results of the randomized clinical trials using SLT

Author, year	SLT IOP red (mmHg) at 6 m—1 y (months)	SLT IOP red (mmHg) end of study (months)	Control group IOP red (mmHg) at 6 m—1 y (months)	Control group IOP red (mmHg) end of study (months)	SLT definition of success	SLT success rate at 6 m—1 y (%) (months)	SLT success rate at end of study (%) (months)
Lai, 2004 [22]	8.0 (12)	8.6 ± 6.7 (60)	7.0 (12)	8.7 ± 6.6 (60)	IOP < 21 mmHg	97	72.41 (60)
Nagar, 2005 [23]	NA	--	NA	--	>20 % IOP red	90° = 34	--
						180° = 65	
						360° = 82 (10)	
Nagar, 2009 [24]	6.2 ± 0.8 (4–6)	--	7.8 ± 0.8 (4–6)	--	>20 % IOP red	75 (4–6)	--
Katz, 2012 [21]	6.3 ± 2.7 (12)	--	7.0 ± 1.8 (12)	--	IOP ≤ 2 mmHg above target IOP + no VF loss ≥ 3 unit	80 (12)	--
Bovell, 2011 [26]	6.0 ± 6.1 (12)	7.4 ± 7.3 (60)	6.0 ± 4.8 (12)	6.7 ± 6.6 (60)	>20 % IOP red	71 (12)	25 (60)
Liu, 2012 [27]	3.8 (12)	1.8 (24)	2.7 (12)	2.8 (24)^a^	def 1: > 20 % IOP red	55 (12)	def 1: 40 (24)^a^
					def 2: IOP < target IOP		def 2: 75 (24)^a^
Rosenfeld, 2012 [28]	4.3 (12)	--	3.23 (12)	--	≥15 % IOP red	75 (12)	--
Kent, 2013 [29]	6.8 (6)	--	7.0 (6)	--	>20 % IOP red	73 (6)	--

*Abbreviations*: *IOP red* = IOP reduction; *m* = months; *y* = year; *NA* = not available; *red* = reduction; *VF* = visual field; *def* = definition; *end of study* = when study was longer than 12 months

^a^Results of Liu et al. [27] at end of study (37 months) were not available

Five studies used ‘more than 20 % reduction of the IOP compared to baseline IOP’ as their definition of success (Lai et al. [22], Nagar et al. [23, 24], Bovell et al. [26], Kent et al. [29]). Their success rates at 6 months to 1 year varied from 55 to 82 %. Again, Liu et al. reported the lowest success rate. This result is in line with trials that claim SLT can be used to lower IOP in normal tension glaucoma, but rarely (in 22 % of patients) creates an IOP reduction of more than 20 % [36]. The success rate of the group that was treated over 90° of TM by Nagar was the lowest recorded; this treatment was considered as insufficient [23]. Lai [22], Katz [21] and Rosenfeld [28] reported success rates ranging from 75 to 97 % after 6 months to 1 year, but used criteria of success that deviated from the others and appear less stringent (IOP less than 21 mmHg [22], IOP less than 2 mmHg above target IOP [21] or more than 15 % IOP reduction [28].

Prediction of success of SLT was examined in several trials and has proven indifferent to: gender, race [23], family history, other glaucoma risk factors, type and severity of OAG, TM pigmentation [19, 37], pseudo-exfoliation [18], number and type of anti-glaucoma medications [22, 38], previous laser [26, 39], phakic or pseudophakic eyes [40], patent iridotomy [41], presence of systemic hypertension or diabetes mellitus [9, 42].

The only variable that predicts a better IOP-lowering effect after SLT is a higher IOP prior to laser [5, 18, 43, 44]. One study found shortened time to failure with increasing age [33], but this was not confirmed by others. Lee et al. [34] also found a significant correlation between both eyes in almost 80 % of treated OAG subjects; success after SLT in one eye correlates with a higher chance of success in the other, which has been confirmed by Shazly et al. [45]. Thinner corneas (CCT <555 μm) also seemed to give better IOP reduction after SLT [46, 47].

### Long term effect

The effect of ALT diminishes over time and the 5-year success rate is reported to be about 50 % [26]. Long-term effectiveness of SLT seems to show similar results. Bovell et al. [26] started with a 71 % success rate at 1 year, which decreased to 52 % at 2 years, 44 % at 3 years, 38 % at 4 years, and 25 % at 5 years. Lai et al. [22] reported a success rate of 97 % 1 year after SLT and 72 % 5 years after SLT, but as noted; their definition of success was less stringent (IOP below 21 mmHg).

The usefulness of SLT and its possible cost-efficient character [12, 48] thus relies upon its repeatability. Hong et al. [39] published one of the first studies on repeat SLT. They examined the effect of a second SLT over 360° in patients who underwent an initial 360° SLT that was successful for more than 6 months but eventually lost efficacy. They recorded no significant difference between treatments and equal success rates after the first and the second SLT. They also compared the group of patients who had a second SLT after 6 months with a group that only needed a second SLT after 12 months and found no differences. They concluded a repeat SLT could be performed as soon as 6 months after a successful first SLT. Ayala et al. [42] treated only 180° of the TM and examined if repeat SLT had better results when it was performed in another area as the first SLT. After a failed SLT, they randomized patients to receive a second SLT in the same or in a different area than the first SLT. This made no difference, which supports the minimal damage theory.

### Side effects of SLT

Redness, discomfort and anterior chamber reaction in the first week after SLT are very common. Nagar et al. [23] recorded discomfort and pain in the first week in 6 % of the patients that underwent 90° SLT, 20 % after 180°, and 39 % after 360°. Anterior chamber reaction/uveitis was present in 31 % of patients after 90° SLT, 41 % after 180°, and 50 % after 360° treatment. These adverse effects are common but much less pronounced than after ALT [49]. Seven of the RCTs used steroids or non-steroidal anti-inflammatory drops after SLT to diminish the side effects (see Table 3). Katz et al. [21] did not mention the use of pre- or post laser drops. Other short lived side effects include headache, photophobia, corneal abrasion, pigment dispersion and subconjunctival haemorrhage [25].

**Table 3:** Side effects mentioned in the randomized clinical trials using SLT

Author, year	Drops administered before SLT	Drops administered after SLT	Redness First week	Discomfort First week	Anterior chamber reaction First week	Definition IOP spike	IOP spike (%)	Other side-effects
Lai, 2004 [22]	apra	apra 1x + pred 4x, 7d	yes	NA	yes	>5 mmHg	10	none
Nagar, 2005 [23]	ameth	dex or keto 4x, 5d	NA	90° = 6 %; 180° = 20 %; 360° = 39 %	90° = 31 %; 180° = 41 %; 360° = 50 %	≥5 mmHg	90° = 9 %; 180° = 16 %; 360° = 27 %	none
Nagar, 2009 [24]	ameth	keto 4x, 5d	NA	NA	NA	NA	NA	NA
Katz, 2012 [21]	NA	NA	NA	NA	NA	NA	NA	none
Bovell, 2011 [26]	apra or brim	pred 4x, 5d	NA	NA	NA	≥6 mmHg	4.5	NA
Liu, 2012 [27]	brim	fluoro 4x, 5d	NA	NA	NA	NA	none	none
Rosenfeld, 2012 [28]	apra	dex 3x, 7d	NA	NA	NA	NA	NA	none
Kent, 2013 [29]	brim + pilo	keto 4x, 5d	NA	NA	NA	>6 mmHg	NA	NA

*Abbreviations*: *NA* = not available; *apra* = 1 % apraclonidine; *pred* = 1 % prednisolone acetate; *ameth* = 1 % amethocaine; *dex* = 0.1 % dexamethasone; *keto* = ketorolac; *brim* = 0.2 or 0.15 % brimonidine; *fluoro* = 0.1 % fluorometholone; *pilo* = 1 % pilocarpine; *4x* = 4 times daily; *5d* = for 5 days; *7d* = for 7 days

#### IOP spike

The transient IOP spike after laser trabeculoplasty is supposed to be dependent upon the energy used per pulse and the total energy administered [50]. Since SLT uses much less energy than ALT, per pulse (0.9 mJ compared to 50 mJ) the IOP-rise is much less common and less pronounced [26–28, 49]. Still, a short increase of more than 5 mmHg above baseline IOP was recorded in 4.5–27 % of patients in three of the RCTs (Lai et al. [22], Nagar et al. [23] and Bovell et al. [26]).

As in ALT [50], a drop of apraclonidine [22, 33, 39, 51–53] or brimonidine [54–56] before and immediately after SLT can be used to prevent the IOP-spike. Seven of the RCTs mentioned the use of apraclonidine, amethocaine, or brimonidine prior to SLT. If IOP elevation did occur, it was treated with anti-glaucoma medication.

#### Corneal changes

Transient corneal thinning and changes in endothelial cell count seem common after SLT, with recovery to normal after one month [57, 58]. In the eight RCTs, no other complications after SLT were noticed. Other studies reported on some rare corneal changes: two cases of transient corneal oedema [59] and one case of corneal decompensation [60] after SLT have been described.

Knickelbein et al. [35] reported 4 cases of corneal hydrops, a condition characterized by acute corneal oedema followed by stromal thinning. Their findings suggest that SLT induces cytokine production and activation of matrix metalloproteinases, which are also involved in destructive inflammatory responses and can enhance collagen degeneration by corneal fibroblasts.

#### Cystoid macular oedema

Two cases of cystoid macular oedema [61, 62] have been reported, but it is suggested that this kind of complication is related to previous complicated lens-surgery. Caution would be needed in patients after compromised cataract surgery. Other very rare complications that have been reported include one case of iritis and choroidal effusion [63] and two cases of hyphema [64] after SLT.

## Conclusions

Robust evidence was presented that there is no significant difference in IOP lowering effect between medication and SLT or between ALT and SLT. At 6 months to 1 year, the success rate of SLT varied between 55 and 82 %. Higher baseline IOP is correlated to higher IOP lowering effect of SLT.

Side effects of SLT are minor. A transient IOP spike is common. Apraclonidine, amethocaine, or brimonidine drops can be administered before and immediately after SLT to counter this IOP rise. More severe side effects like corneal or macular oedema, peripheral anterior synechiae, hyphema, severe uveitis or persistent IOP rise are very rare.

### Topics to be addressed in the future

Further investigation is needed to demonstrate if SLT can be repeated enough to maintain IOP under control for decades on end. Possibly, the future of SLT lies in a yearly application of a smaller number of spots, as is currently under investigation by Gandolfi et al. (Vienna, SOE meeting, 2015).

In conclusion, SLT may be introduced into glaucoma management algorithms in two ways. First, as a primary treatment to patients with OAG, comparable in efficacy to medication. Second, as a treatment alternative for patients not controlled with maximally tolerated medication, before invasive surgery.
